# Platelet miRNAs: differential expression in coronary artery disease and associations with course of left ventricular systolic function

**DOI:** 10.1186/s12872-023-03362-0

**Published:** 2023-07-12

**Authors:** Andreas Goldschmied, Bernhard Drotleff, Stefan Winter, Elke Schaeffeler, Matthias Schwab, Meinrad Gawaz, Tobias Geisler, Dominik Rath

**Affiliations:** 1 Department of Cardiology, University Hospital Tübingen, Otfried-Müller Str. 10, 72076 Tübingen, Germany; 2 European Molecular Biology Laboratory, Heidelberg, Germany; 3 University of Tübingen, Tübingen, Germany; 4 Dr. Margarete Fischer-Bosch-Institute of Clinical Pharmacology, Stuttgart, Germany; 5 Departments of Clinical Pharmacology, Pharmacy and Biochemistry, University of Tübingen, Tübingen, Germany

**Keywords:** Platelet microRNA, Coronary artery disease, Myocardial infarction, MiRNA 103a-3p, MiRNA 155-5p, Left ventricular ejection fraction

## Abstract

**Background:**

MicroRNAs are paramount in post transcriptional gene regulation. We investigated platelet miRNAs in patients with CAD and examined potential associations with course of left ventricular ejection fraction (LVEF%).

**Materials and methods:**

In a first cohort, 62 MiRNAs were measured in platelets of 100 patients suffering from CAD. Expression profiles of individuals with chronic coronary syndrome (CCS) and MI were compared (CCS n = 67, MI n = 33). Also, associations between miRNA profiles and change in left ventricular ejection fraction (LVEF%) were investigated. In a second cohort of patients suffering from CCS (n = 10), MI (n = 11) or no CAD (n = 13), we measured miRNA expression in platelets, platelet supernatant and serum. This was carried out before and after in vitro platelet activation with CRP.

**Results:**

Platelet miRNAs 103a-3p and 155-5p demonstrated higher expression in patients with CCS then in individuals with MI. Furthermore, multiple miRNAs were significantly higher expressed in matched controls compared to MI patients. 8 miRNAs showed higher expression in patients with improving LVEF% after a 1-year follow-up. In our second cohort, we found higher concentrations of 6 miRNAs in the platelet supernatant of patients with CCS, MI and no CAD after in vitro platelet activation. Most of these miRNAs showed a higher abundance in serum of MI patients as compared to CCS.

**Conclusion:**

Several miRNAs show higher expression in platelets of CCS compared to MI. After in vitro platelet activation, a release of multiple miRNAs out of the thrombocyte was observed. Furthermore, upregulation of serum miRNAs was found in MI patients when compared to CCS patients and individuals without CAD. Hence, platelets could present a source of upregulated circulating miRNAs in MI and additionally affect course of LVEF%.

**Supplementary Information:**

The online version contains supplementary material available at 10.1186/s12872-023-03362-0.

## Introduction

MicroRNAs (miRNAs) are short endogenous RNAs that play important roles in gene regulation [1]. They are non-coding, approximately 22 nucleotides in length and mediate gene activity by guiding argonaute proteins to the 3´ untranslated region of mRNAs, subsequently blocking translation and causing mRNA degradation. MiRNAs are stable in vivo and can be detected in plasma, serum and tissue [2].

MiRNAs are known to play key roles in cardiovascular disease and especially atherosclerosis [3]. In several observational studies, miRNA expression profiles were compared between patients suffering from coronary artery disease (CAD) and controls or between those with acute coronary syndrome (ACS) and chronic coronary syndrome (CCS) [4]. Several potential prognostic and diagnostic markers have been identified [5–10].

However, there are view studies investigating changes in the platelet miRNA expression profile in CAD. Platelets are well known to play a central role in thrombo-inflammation and cardiovascular disease. Approximately 30% of genes are abundant in platelets at mRNA level. Possessing the necessary components for mRNA translation, platelets may synthesize proteins despite their lack of a nucleus, a pre-requisite for transcription. As in other cells, miRNAs possess regulatory functions at the translational level in platelets [11, 12]. Moreover, platelets may rely on miRNA as a post transcriptional mechanism for regulating gene expression [13].

The origin of miRNAs detected in human plasma and serum is not clear yet. Data suggests that parts of the circulating miRNAs travel dependent on exosomes [2, 14]. Platelets serve as a possible source of circulating miRNAs. Several studies demonstrate that miRNA packaging in platelet microparticles is promoted by platelet activation and can mediate gene expression in cells like macrophages, smooth muscle cells and endothelial cells via miRNA as a paracrine transmitter [15–19]. Several of these miRNAs are also linked to the development and pathogenesis of CAD. For example, miRNA 22 has been demonstrated to promote coronary artery disease by targeting MCP-1 and treatment with extracellular vesicles containing miRNA 185 promotes atherosclerosis in mice [20, 21].

However, little is known about changes in the platelet miRNA expression profile in CAD. In this study, we compared miRNA expression profiles in resting platelets, activated platelets and serum from patients suffering from myocardial infarction (MI), CCS and those without CAD. Furthermore, we investigated associations between course of systolic left ventricular ejection fraction (LVEF%) and platelet miRNA expression levels.

## Materials and methods

### Study population

In this prospective cohort study, we enrolled 134 individuals in two separate study cohorts. Patients were admitted to the department of Cardiology and Angiology at the University Hospital of Tuebingen, Germany from May to July of 2017.

In the first cohort (n = 100), 33 patients presented with acute MI, 67 patients were diagnosed with CCS. In the second cohort (n = 34), 11 patients presented with acute MI, 10 patients with CCS and 13 patients without CAD. MI was defined using the universal definition of myocardial infarction according to the 2015 and 2017 ESC guidelines [22, 23], CCS was defined using the 2013 ESC guidelines [24]. The individuals either presented for a scheduled coronary angiography or were transferred for invasive diagnostics and therapy to the catheter laboratory via the emergency room.

In the first cohort, 60 mL of arterial blood were drawn periprocedurally via the catheter sheet. In the second cohort, 27,5 mL of venous blood were drawn from a cubital vein the day after coronary intervention (16–25 h).

All samples were directly transferred to our laboratory for platelet isolation and further measurements. Inclusion criteria were age > 18 years and invasively diagnosed CAD for the MI and CCS groups in cohort 1 and 2.

MiRNA measurements in cohort 2 were based on the results of cohort 1 (described in more detail in the section “sample preparation and measurement”) but the two cohorts have been studied separately.

All patients gave written informed consent. The study complies with the declaration of Helsinki and good clinical practice guidelines and was approved by the responsible ethics committee (270/2011B01) and (238/2018BO2).

### Sample preparation and measurement

Blood was pooled in a ratio of 1:4 with Acid-Citrate-Dextrose (ACD) anticoagulant buffer and platelets were isolated as previously described by Burkhart et al. (full protocol in the supplementary) [25]. Platelet pellets were labeled and stored at -80 °C.

In cohort 1, total RNA including miRNA was extracted from platelet pellets using mirVana miRNA isolation kit (Applied Biosystems, Thermo Fisher Scientific, USA) following the manufacturer’s protocol and stored at − 80 °C. MiRNA selection for quantification by real-time PCR was based on literature data related to cardiovascular disease [26, 27]. In brief, miRNAs were reversely transcribed according to the manufacturer’s protocol using TaqMan microRNA Reverse Transcription Kit (Applied Biosystems, Thermo Fisher Scientific, USA) and pooled individual stem-loop reverse transcription primers were included in the pre-developed TaqMan miRNA assays (Applied Biosystems, Thermo Fisher Scientific, USA).

Next, a pre-amplification reaction was performed to improve the sensitivity of miRNA quantification, using TaqMan PreAmp Master Mix (Applied Biosystems, Thermo Fisher Scientific, USA) and pooled pre-developed TaqMan miRNA assays (Applied Biosystems, Thermo Fisher Scientific, USA). Pre-amplification PCR was run according to the manufacturer’s protocol. The resulting PCR product was diluted 1:5 with suspension buffer (Teknova AS, Norway) and stored at − 20 °C until quantification by real-time PCR. The miRNA expression levels were quantified by real-time PCR using TaqMan® Universal Master Mix II (no UNG) and pre-developed TaqMan miRNA assays (Applied Biosystems, Thermo Fisher Scientific, USA) on a real-time PCR BioMark system (Fluidigm Corporation, USA) following the manufacturer’s protocol. Relative levels of miRNA expression were calculated by normalization to expression levels of miRNA 24-3p, miRNA 320a and miRNA-451a (delta-delta CT value). Based on a literature study, 62 miRNAs with known associations to the cardiovascular system were measured [26, 27].

In cohort 2, miRNeasy miRNA isolation kit (Qiagen, Netherlands) was used to extract total RNA from serum, the platelet pellet and platelet supernatant following the manufacturer’s protocol. Samples were stored at − 80 °C. MiRNAs were then reversely transcribed according to the manufacturer’s protocol using miRCURY LNA RT Kit (Qiagen, Netherlands) and stored at -20 °C until quantification by real-time PCR. MiRNA expression levels were quantified by real-time PCR miRCURY LNA SybrGreen assays (Qiagen, Netherlands) on a real-time PCR light cycler LC480 system (Roche, Switzerland).

MiRNA expression in the supernatant and serum was calculated by normalization to expression levels of cel-miR-39. To calculate relative levels of miRNA in platelets, normalization to expression levels of U6 snRNA was carried out. Target miRNAs for cohort 2 were chosen based on the analyzes of the MI group compared to the CCS and matched control (MC) group from cohort 1and whether the individual miRNA could be detected in the platelet supernatant at reasonable levels. This yielded 6 miRNAs which were measured in cohort 2.

### In vitro platelet activation

In vitro platelet activation with CRP for cohort 2 was carried out as previously described [19]. Before activation, 2mM CaCl_2_ solution was added to the platelet suspension. Afterwards, platelets were either incubated with CRP (CRP-XL solution, 2 µg/mL) for 10 min at 37 °C or kept under resting conditions. After this incubation period, the samples were centrifuged at 2500xg for 15 min to pellet the platelets, removed cell debris and collect platelet supernatant. MiRNAs were measured in activated and resting platelets as well as the platelet supernatant with and without platelet activation (see section above). Platelet activation was confirmed by flow cytometry analyses of CD 62P denoting degranulation from α-granules.

### Statistical analyses

Data processing and statistical evaluation were conducted with Excel 2019 (Microsoft, Redmond, WA, USA), SPSS Statistics 23 (IBM, Armonk, NY, USA), Origin 2019 (OriginLab, Northampton, MA, USA), and RStudio 1.2.1335 (R version R-3.5.1, R Foundation for Statistical Computing, Vienna, Austria) [28].

In cohort 1, missing miRNA data (1.48% of values) were imputed using a random forest-based method [29]. The resulting data was used to compare miRNA expression between groups (ACS vs. CCS, ∆ LVEF%>1 vs. ∆ LVEF%<1). Calculation of relative miRNA expression profiles for the whole population was based on non-imputed data. For hypothesis testing Mann-Whitney U tests were applied. Fold changes between experimental groups were calculated via the Hodges-Lehmann estimator (i.e. pseudomedian). To account for confounding factors which could alter the miRNA expression profile, 28 individuals from the CCS group were matched to 28 patients from the MI group (suitable matches were lacking for 5 MI patients). Matching factors included age (0 to 6 years), sex, diabetes and history of chronic kidney failure. The resulting data was used to compare miRNA expression between MI and matched controls (MC). Missing miRNA data (1.15% for the matched control dataset) were also imputed using the same random forest-based method. For hypothesis testing of the matched dataset, Wilcoxon signed rank tests were used. Fold changes for this dataset were also calculated via the Hodges-Lehmann estimator (i.e. pseudomedian).

In the second cohort, missing Data (3.83% of values for miRNA, 8.82% of values for flow cytometry analyses) were not imputed. For hypothesis testing of paired variables (resting vs. activated platelet supernatant and resting vs. activated platelets), paired t-tests were used. For comparing miRNA expression profiles across groups, Mann-Whitney U tests were used.

Concerning the baseline characteristics, chi-square tests and McNemar tests were applied to compare dichotomous variables whereas Mann-Whitney U, Kruskal-Wallis and Wilcoxon signed rank tests were used for metric variables. All statistical tests were two-sided and statistical significance level was defined as 5%. No adjustment for multiple testing was performed.

### Course of LVEF%

If available, periprocedural echocardiography and/or ventriculography were analyzed and LVEF% was determined in the patient collective of cohort 1. One year after study inclusion, the electronic database was searched for possible readmissions and follow-up echocardiography and/or ventriculography. Eligibly image studies were revisited and LVEF% was measured. We calculated course of LVEF% by dividing initial ejection fraction by ejection fraction on follow up. This yielded three groups: Patients with improved LVEF% (> 1), patients with deteriorated LVEF% (< 1) and patients with no change in LVEF% (= 1).

## Results

Baseline characteristics of cohort 1 are shown in Table 1. As mentioned in the “Materials and Methods” section, miRNA profiles are altered by numerous factors. Therefore, we matched patients suffering from CCS (matched controls or “MC”) to MI patients. We chose age (age difference of 0 to 6 years), sex, diabetes and history of chronic kidney failure as matching variables. For n = 5 of the n = 33 MI patients, suitable matches within the CCS group were lacking. As a consequence, analyses of matched data were performed based on the remaining n = 28 pairs only.

**Table 1:** Baseline Characteristics of cohort 1 Summary statistics for numeric variables are presented as pseudomedian (range). Mann-Whitney U tests were applied to compare distributions in the overall population, Wilcoxon signed rank tests were used for the matched population Chi-square tests were applied to compare dichotomous variables in the overall population, McNemar tests were applied to compare dichotomous variables in the matched population

	Total (n = 100)	MI (n = 33)	CCS (n = 67)	p-value	MI (n = 28)	MC (n = 28)	p-value
Gender (male%)	76 (76%)	23 (70%)	53 (79%)	p = 0.300	22 (79%)	22 (79%)	p = 1
Age (years)	72.5 (45)	76 (54)	71 (41)	p = 0.800	74 (39)	74 (33)	p = 0.828
CV Risk Factors							
Hypertension	76 (78%)	22 (67%)	55 (83%)	p = 0.060	19 (68%)	24 (86%)	p = 0.180
Diabetes	29 (30%)	10 (30%)	19 (29%)	p = 0.876	8 (29%)	8 (29%)	p = 1
Previous MI	22 (22%)	4(12%)	18 (27%)	p = 0.094	3(11%)	6(21%)	p = 0.508
GFR (ml/min)	73.3 (240)	73.9 (22)	72.8 (171)	p = 0.822	72.3 (93)	76.7 (140)	p = 0.662
Smoking	19 (20%)	6 (18%)	14 (21%)	p = 0.723	6 (21%)	6 (14%)	p = 0.688
Hyperlipidemia	42 (42%)	9 (27%)	33 (49%)	p = 0.310	8 (29%)	14 (50%)	p = 0.263
Laboratory values							
Troponin positive	33 (33%)	33 (100%)	0 (0%)	**p < 0.001**	28 (100%)	0 (0%)	**p < 0.001**
CK (U/l)	113.0 (3642)	145 (3604)	81 (655)	**p < 0.001**	134.5 (3601)	116 (629)	p = 0.073
CRP (mg/dl)	0.33 (23.28)	0.5 (23.28)	0.19 (8.45)	**p = 0.019**	0.46 (23.23)	1.10 (8.45)	p = 0.166
Medication on Admission							
ASA	54 (57%)	13 (45%)	41 (63%)	p = 0.098	10 (42%)	18 (64%)	p = 0.424
Clopidogrel	8 (9%)	0 (0%)	8 (12%)	**p = 0.048**	0 (0%)	4 (14%)	p = 0.248
Prasugrel	5 (5%)	0 (0%)	5 (8%)	p = 0.125	0 (0%)	3 (11%)	p = 0.248
Ticagrelor	12 (13%)	2 (7%)	10 (15%)	p = 0.255	2 (8%)	7 (25%)	p = 0.688
Statins	67 (71%)	14 (48%)	53 (82%)	**p = 0.001**	12 (50%)	25 (89%)	**p = 0.006**
ACE Inhibitors	41 (43%)	10 (35%)	31 (48%)	p = 0.233	9 (38%)	14 (50%)	p = 0.607
ARBs	25 (27%)	6 (21%)	19 (29%)	p = 0.387	4 (17%)	9 (32%)	p = 0.508
Beta Blockers	56 (60%)	14 (48%)	42 (65%)	p = 0.136	11 (46%)	20 (71%)	p = 0.302
Diuretics	39 (42%)	12 (41%)	27 (42%)	p = 0.988	10 (42%)	12 (43%)	p = 1
Coronary Artery Disease							
MI	33 (33%)	33 (100%)	0 (0%)	**p < 0,001**	28 (100%)	0 (0%)	**p < 0,001**
CCS	67 (67%)	0 (0%)	67 (0%)	**p < 0,001**	0 (0%)	28 (100%)	**p < 0,001**
LVEF%							
LVEF% admission	55 (52.5)	50 (45)	55 (52.5)	**p = 0.041**	52.5 (40)	55 (40)	p = 0.084
LVEF% follow up	55 (40)	55 (30)	57.5 (40)	p = 0.414	55 (30)	57.5 (25)	p = 0.628

Overall, the expression levels of the 62 measured miRNAs within cohort 1 appeared to be rather heterogenic and no global trend between CCS and MI patients could be observed. They are listed in supplementary Tables 1, ordered by their average expression based on all 100 patients. Supplementary Fig. 1 shows a heatmap of miRNA expression profiles of cohort 1.

To observe platelet miRNA concentrations upon in vitro platelet activation, cohort 2 was recruited. Baseline characteristics for this second study cohort are shown in Table 2.

**Table 2:** Baseline Characteristics of cohort 2 Summary statistics for numeric variables are presented as pseudomedian (range). Chi-square tests were applied to compare dichotomous variables, Kruskal Wallis tests were applied to compare metric variables

	Total (n = 34)		MI (n = 11)		CCS (n = 10)		no CAD (n = 13)	p-value
Gender (male%)	19 (56%)		9 (82%)		6 (60%)		4 (31%)	**p = 0.041**
Age (years)	63.5 (65)		60 (40)		72 (45)		53 (64)	**p = 0.034**
CV Risk Factors								
Hypertension	22 (65%)		10 (91%)		9 (90%)		3 (23%)	**p < 0.001**
Diabetes	6 (18%)		3 (27%)		2 (20%)		1 (8%)	p = 0.444
Previous MI	1 (3%)		0 (0%)		1 (10%)		0 (0%)	p = 0.290
GFR (ml/min)	82.5 (77)		95.3 (64)		81.8 (58)		75.5 (43)	p = 0.136
Smoking	6 (18%)		3 (27%)		1 (10%)		2 (15%)	p = 0.563
Hyperlipidemia	9 (27%)		4 (36%)		4 (40%)		1 (8%)	p = 0.146
Laboratory values								
Troponin positive	11 (32%)		11 (100%)		0 (0%)		0 (0%)	**p < 0.001**
CK (U/l)	126 (3082)		278 (3016)		76 (78)		108.5 (197)	**p < 0.001**
CRP (mg/dl)	0.29 (5.4)		2.15 (5.3)		0.95 (1.9)		0,14 (0.72)	**p = 0.004**
Medication on Admission								
ASA	20 (59%)		9 (82%)		10 (100%)		1 (8%)	**p < 0.001**
Clopidogrel	13 (38%)		4 (36%)		1 (10%)		1 (8%)	**p < 0.001**
Prasugrel	6 (18%)		6 (55%)		0 (0%)		0 (0%)	**p < 0.001**
Ticagrelor	2 (6%)		1 (9%)		1 (10%)		0 (0%)	p = 0.542
Statins	20 (59%)		10 (91%)		9 (90%)		1 (8%)	**p < 0.001**
ACE Inhibitors	4 (12%)		1 (9%)		3 (30%)		0 (0%)	p = 0.093
ARBs	15 (44%)		10 (91%)		4 (40%)		1 (8%)	**p < 0.001**
Beta Blockers	12 (35%)		9 (82%)		3 (30%)		0 (0%)	**p < 0.001**
Diuretics	7 (21%)				4 (40%)		1 (8%)	p = 0.186
FACS								
Delta CD62	1482 (2639)		613 (1621)		1244 (2384)		2116 (2050)	**p < 0.001**
LVEF%								
LVEF% admission	55 (30)		55 (15)		55 (30)		55 (0)	p = 0.307

### Differences in miRNA expression between MI and CCS

**Fig. 1:**
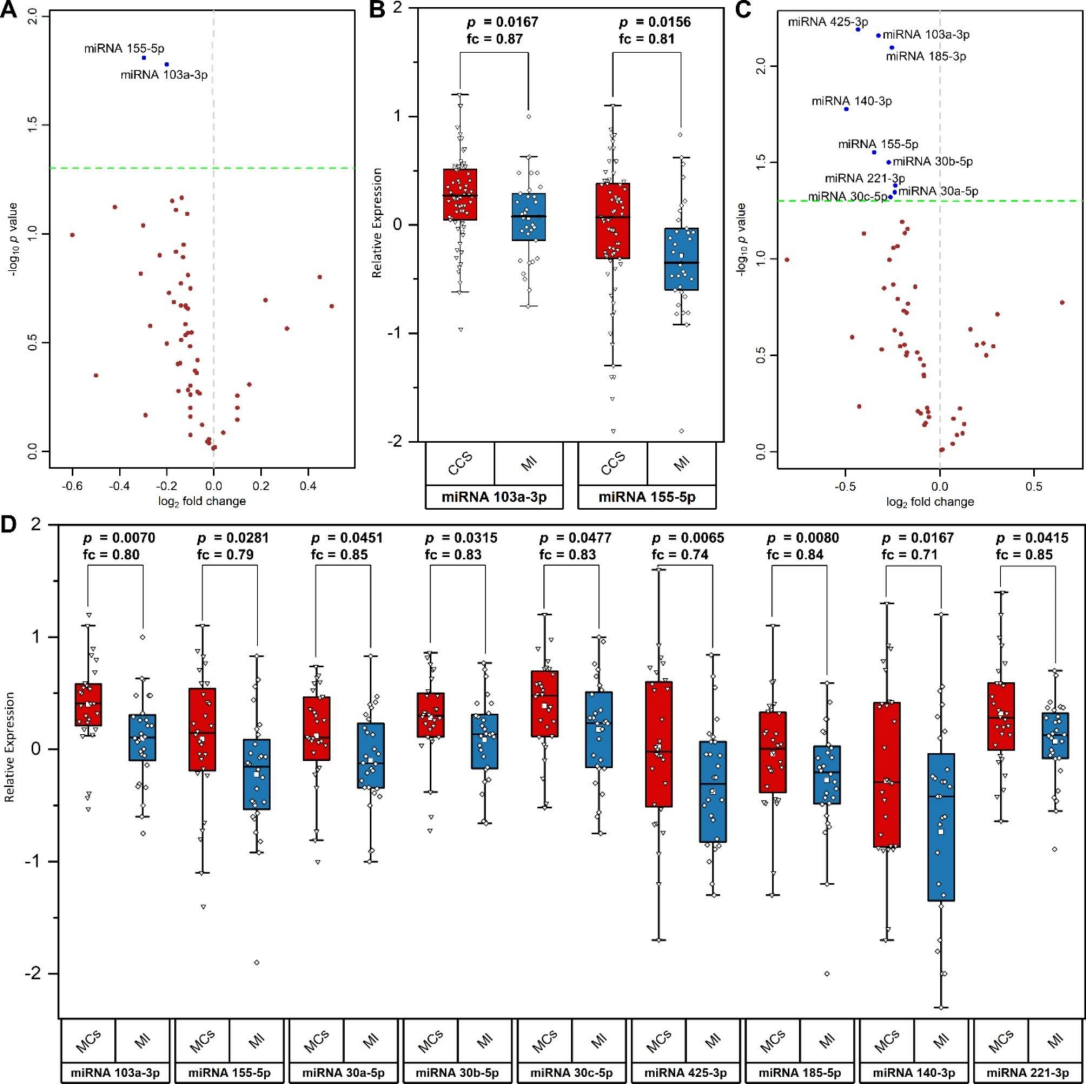
Comparison of individual miRNA expression levels between MI (n = 33) and CCS (n = 67) patients and matched MI (n = 28) and CCS (MC; n = 28) patients (**a**) Volcano plot related to the analysis of individual miRNA expression levels between CCS (n = 67) and MI (n = 33). The x-axis presents fold change on a log_2_ scale (positive values represent a higher expression of miRNAs in CCS compared to MI), the y-axis displays -log_10_ p-values (dots above the horizontal line (p = 0.05) represent significantly differentially expressed miRNAs); (**b**) Box plots comparing expression levels of miRNA 103a-3p and miRNA 155 in MI (n = 33) and CCS (n = 67); (**c**) Volcano plots related to the analysis of individual miRNA expression levels between MCs (n = 28) and MI (n = 28). The x-axis presents fold change on a log_2_ scale (positive values represent a higher expression of miRNAs in MCs compared to MI), the y-axis displays -log_10_ p-values (dots above the horizontal line (p = 0.05) represent significantly differentially expressed miRNAs) (**d**) Box plots comparing expression levels of miRNA 103a-3p, 155-5p, 30a-5p, 30b-5p, 30c-5p, 425-3p, 185-5p, 140-3p and 221-3p in patients with MI (n = 28) and MCs (n = 28)

As seen in Fig. 1a/b, miRNAs 103a-3p and 155-5p were significantly lower expressed in MI patients (n = 33) as compared to CCS (n = 67).

The subgroup analyses with matched controls (MC) confirmed our principle whole-cohort analysis with lower expression of miRNAs 103a-3p and 155-5p in MI. Additionally, miRNAs 30a-5p, 30b-5p, 30c-5p, 185-5p, 140-3p, 221-3p and 425-3p were significantly lower expressed in MI patients compared to matched controls (MC) (Fig. 1c/d).

### Associations of miRNA expression and course LVEF%

Course of LVEF% was determined in 48 patients from cohort 1. Individual miRNA expression levels were compared between patients with improved or worsened LVEF% (Δ < 1 or > 1) over the span of one year. Other influence factors that could alter course of LVEF% are infarct size and severity of CAD. However, neither extent of CAD (one versus two versus three-vessel disease) nor levels of troponin or creatine kinase varied significantly between patients with improved, worsened or stable LVEF%.

MiRNAs 21-5p, 23b-3p, 27a-3p, 29a-3p, 29b-3p, 92a-3p, 221-3p and the miRNA precursor let-7c-5p showed significantly higher baseline expression profiles in the subgroup with improved vs. worsened LVEF% over time (supplementary Fig. 2a). Exemplary boxplots for differently expressed miRNAs 23b-3p and 29b-3p are shown in supplementary Fig. 2b.

### Platelet miRNA shift upon activation

**Fig. 2:**
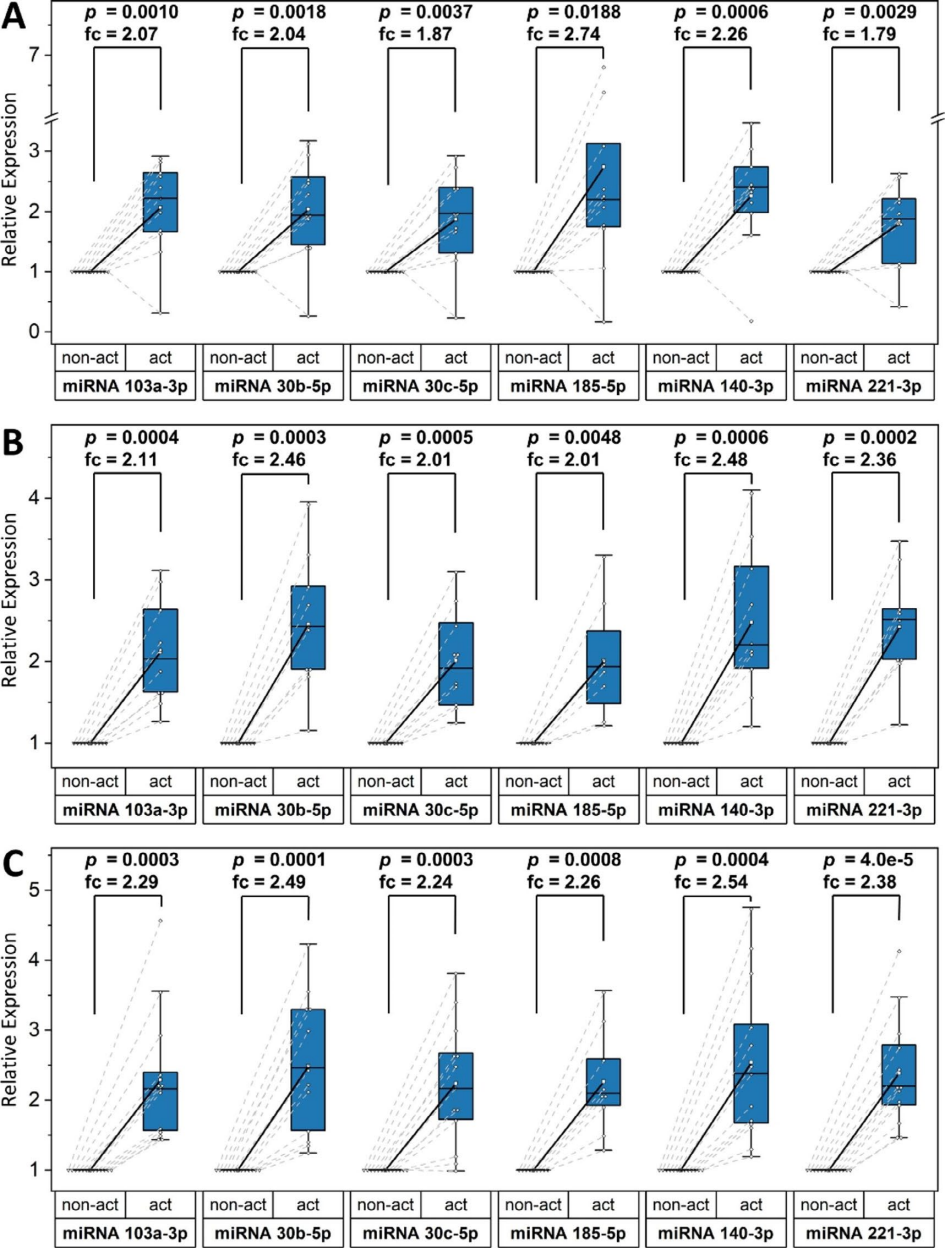
Comparison of miRNA concentrations in platelet supernatant before and after activation with CRP. The y-axis represents relative miRNA expression, p-values and corresponding fold changes (fc) are indicated on top of the boxes (**a**) Box plots comparing expression levels of miRNAs 103a-3p, 30b-5p, 30c-5p, 185-5p, 140-3p and 221-3p in platelet supernatant before (non-act) and after (act) in vitro platelet activation in patients with MI (n = 11); (**b**) Box plots comparing expression levels of miRNAs 103a-3p, 30b-5p, 30c-5p, 185-5p, 140-3p and 221-3p in platelet supernatant before (non-act) and after (act) in vitro platelet activation in patients with CCS (n = 10); (**c**) Box plots comparing expression levels of miRNAs 103a-3p, 30b-5p, 30c-5p, 185-5p, 140-3p and 221-3p in platelet supernatant before (non-act) and after (act) in vitro platelet activation in individuals with no CAD (n = 13)

To analyze miRNA shift upon activation, cohort 2 was recruited. As shown in Fig. 2, all miRNA concentrations (30b-5p, 30c-5p, 103a-5p, 140-3p, 185-5p and 221-3p) in the platelet supernatant increased significantly upon in vitro platelet activation in the MI-, CCS- and no CAD group, respectively. Even though fold change for miRNAs 30b-5p, 30c-5p and 221-3p was slightly higher in the no CAD group, there was no statistically significant difference between the groups. Fold change was independent of antiplatelet medication or platelet activation levels.

The change in platelet miRNA concentrations after in vitro activation is demonstrated in supplementary Fig. 3. Here, we could observe only a significant downregulation of miRNA 103a-5p and 221-3p within the CCS cohort. In this second cohort, concentrations of miRNAs 30b-5p, 30c-5p, 103a-5p, 140-3p, 185-5p and 221-3p in resting platelets were not significantly different between ACS, CCS and no CAD.

### Serum miRNA concentrations

**Fig. 3:**
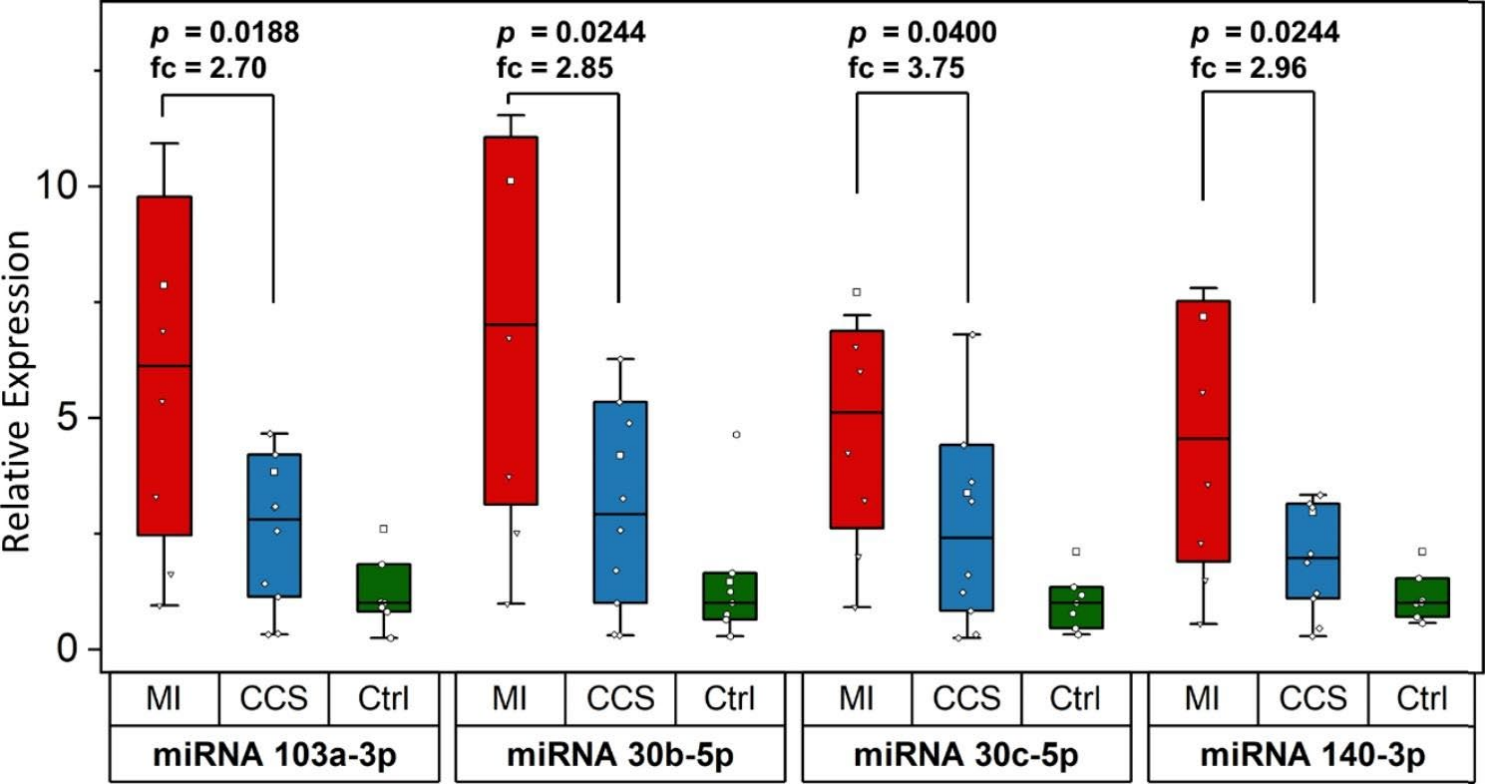
Box plots comparing expression levels of miRNA 103a-3p, 30b-5p, 30c-5p and 140-3p in serum of patients with MI (n = 9), CCS (n = 9) and no CAD (n = 7). The y-axis represents relative miRNA expression, p-values and corresponding fold changes (fc) between the MI and CCS cohort are indicated on top of the boxes

MiRNAs 30b-5p, 30c-5p, 103a-5p, 140-3p, 185-5p and 221-3p were measured in patients’ serum in cohort 2. MiRNAs 30b-5p, 30c-5p, 103a-5p and 140-3p showed significantly higher concentrations in the MI cohort compared to CCS and are displayed in Fig. 3.

## Discussion

In this study, we compared platelet miRNA expression profiles for 62 miRNAs between patients with MI and CCS. Additionally, we compared 6 selected miRNAs in patients with MI, CCS and no CAD in serum, platelets and platelet derived supernatant after in vitro platelet activation. The major findings of the present study are: [1] miRNAs 103a-3p and 155-5p showed significantly lower expression levels in platelets from MI compared to CCS patients [2]. Additionally, miRNAs 30a-5p, 30b-5p, 30c-5p, 185-5p, 140-3p, 221-3p and 425-3p showed significantly lower expression levels in MI patients compared to matched controls [3]. MiRNAs 30b-5p, 30c-5p, 103a-5p, 140-3p, 185-5p and 221-3p are released from platelets upon in vitro activation with CRP [4]. MiRNAs 30b-5p, 30c-5p, 103a-5p and 140-3p showed a significantly higher expression in serum of patients suffering from MI compared to CCS [5]. MiRNAs 21-5p, 23b-3p, 27a-3p, 29a-3p, 29b-3p, 92a-3p, 221-3p and the miRNA precursor let-7c-5p levels at baseline correlated significantly with course of LVEF% in a 1-year follow-up.

As for miRNAs 103a-3p and 155-5p, supplementary Table 2 summarizes their suggested effects on cardiovascular disease in the literature.

MiRNA 103 is believed to play a role in cardiomyocyte necrosis and atherosclerosis through activation of FADD and by suppression of KLF4 [30, 31]. MiRNA 155 may promote atherosclerosis via activation of endothelial cells and could inhibit megakaryopoiesis [32, 33]. Clinical data on miRNA 155 is however contradictory. Downregulation in peripheral blood mononuclear cells (PBMCs) of ACS as compared to CCS patients and downregulation in plasma of CAD patients compared to healthy controls have been reported [7, 34]. On the other hand, data suggest that plasma upregulation of miRNA 155 is associated with coronary slow flow [35]. Literature on miRNAs 30b-5p, 30c-5p, 103a-5p, 140-3p, 185-5p 221-3p and 425-3p shows regulatory properties in atherosclerosis as well as cardiomyocyte apoptosis and necrosis [21, 36–42].

As mentioned previously, the lack of a cell nucleus in platelets and their inability to execute transcription render them prone to rely on miRNAs to regulate protein synthesis [13]. Data show that platelet activation promotes exosome- and microparticle-depended release of miRNAs. Paracrine effects in smooth muscle cells, macrophages and endothelial cells have been observed [2, 14–18]. Gidlöf et al. demonstrated platelet release and endothelial cell uptake of miRNA 185 in MI [15].

We could currently demonstrate miRNA release from platelets upon activation with CRP for miRNAs 30b-5p, 30c-5p, 103a-5p, 140-3p, 185-5p and 221-3p. Those paracrine effects may explain our findings showing lower expression levels of potentially pro-atherosclerotic miRNAs in platelets of patients suffering from MI. Platelets might serve as a source for circulatory miRNAs which are released upon activation and mediate atherosclerosis and cardiomyocyte necrosis through the mechanisms described in the literature. This hypothesis is supported by higher serum miRNA 30b-5p, 30c-5p, 103a-5p and 140-3p concentrations in MI as compared to CCS patients. Since blood in the second cohort was drawn the day after coronary intervention, intrinsically activated platelets might have already released miRNAs into the circulation.

However, to what extent platelets really contribute to those elevated concentrations of serum miRNAs under physiological circumstances, remains unclear. We could observe a significant reduction in platelet miRNAs 103a-5p and 221-3p after in vitro activation in the CCS group. This finding may indicate that only a small amount of platelet miRNAs is excreted upon activation and that elevated circulating miRNA levels could also originate from other sources. In fact, evidence from animal studies suggests that circulating miRNA 140-3p in MI originates mostly from monocytes and circulating endothelial cells [43]. Furthermore, we were not able to measure sufficient miRNA-155 levels in the platelet supernatant. Hence, we did not include this miRNA into the analyses of our second cohort. Reduced levels of resting platelet miRNAs 30b-5p, 30c-5p, 103a-5p, 140-3p, 185-5p and 221-3p in MI compared to CCS could not be confirmed in the second cohort probably due to low sample size, different timing in sample acquisition and different donor vessels (arterial vs. venous blood).

In the current study, we cannot assess the molecular mechanisms of platelet miRNA release upon activation. However, the effect seems to occur independently of antiplatelet medication and degree of platelet activation.

Furthermore, we investigated possible associations of platelet miRNAs with course of LVEF% in patients with MI and CCS. 8 miRNAs showed higher baseline expression levels in individuals with an improvement of LVEF% over time. Multiple studies have investigated the pathophysiological and prognostic role of circulating miRNAs in both acute and chronic systolic heart failure [44]. To our knowledge, this is the first study to investigate platelet miRNAs as possible prognostic factors for course of left ventricular systolic function. Several of the differentially expressed miRNAs in the current study have already been associated with heart failure. MiRNA 27a-3p levels are increased in patients with acute heart failure and plasma miRNA 29a-3p and 92a-3p levels increased after treatment of patients with chronic heart failure with a cardiac resynchronization device [45, 46]. Plasma miRNA-221 is decreased in individuals with systolic heart failure compared to healthy controls. Furthermore, miRNA-221 may help in differentiating between heart failure with reduced and preserved LVEF% [47]. Finally, high serum miRNA-21 levels are associated with systolic heart failure [48].

## Conclusion

MiRNA 103a-3p and miRNA 155-5p showed lower expression levels in platelets of patients with MI compared to CCS. In addition to miRNAs 103a-3p and 155-5p, miRNA 30a-5p, 30b-5p, 30c-5p, 185-5p, 140-3p, 221-3p and 425-3p levels were reduced when comparing MI patients to MCs. We were able to demonstrate platelet miRNA excretion upon platelet activation for miRNAs 30b-5p, 30c-5p, 103a-5p, 140-3p, 185-5p and 221-3p. In serum, miRNAs 30b-5p, 30c-5p, 103a-5p and 140-3p showed significantly higher concentrations in the MI cohort compared to CCS. Therefore, platelets are likely a source for circulating miRNAs in MI.

Finally, 8 miRNAs were associated with course of LVEF% in a 1-year follow-up. Therefore, certain miRNAs may be of use for identifying patients who may benefit from an intensified heart failure therapy.

### Limitations

Our study has several limitations. Due to the observational nature of the study, we cannot adjust for all confounders affecting miRNA expression like cardiovascular risk factors or medication and cannot give explanations for the molecular mechanisms which might cause miRNA release. Also, we did not adjust for all confounders that might influence course of LVEF%.

Furthermore, our study collective is heterogeneous. Even though all patients suffered from invasively diagnosed CAD, not all individuals received PCI (percutaneous coronary intervention). In addition, the MI cohort consisted of both patients with type I and type II MI.

Due to limited follow up data, the sample size for investigating effects on the course of LVEF% is small. Also, we did not adjust for multiple comparisons in our statistical analysis. Finally, LVEF% at admission varied considerably. Limitations of the second cohort include a reduced sample size and incomplete data on flow cytometry and serum miRNA measurements.

## Electronic supplementary material

Below is the link to the electronic supplementary material.

Supplementary figure 1: Heatmap of miRNA expression profiles

Supplementary figure 2: Comparison of individual miRNA expression levels stratified according to Δ LVEF%

Supplementary figure 3: Comparison of miRNA concentrations in platelets before and after activation with CRP.

Supplementary table 1: 62 selected miRNAs

Supplementary table 2: Significantly altered miRNAs and suggested cardiovascular effects

Supplementary: Platelet isolation Protocol

## Data Availability

The datasets used and/or analysed during the current study are available from the corresponding author on reasonable request.
