# Supplementary material for: Platelet miRNAs: differential expression in coronary artery disease and associations with course of left ventricular systolic function
Source: BMC Cardiovasc Disord. 2023 Jul 12;23:348. doi: 10.1186/s12872-023-03362-0 (PMC10339596; doi:10.1186/s12872-023-03362-0)

**Platelet miRNAs: Differential expression in coronary artery disease and associations with course of left ventricular systolic function**

Andreas Goldschmied^1^, Bernhard Drotleff^2^, Stefan Winter^3,4^, Elke Schaeffeler^3,4^, Matthias Schwab^4,5^, Meinrad Gawaz^1^, Tobias Geisler^1^* Dominik Rath^1*^

^1^ Department of Cardiology, University Hospital Tübingen, Tübingen, Germany

^2^ European Molecular Biology Laboratory, Heidelberg, Germany

^3^ University of Tübingen, Tübingen, Germany

^4^ Dr. Margarete‐Fischer‐Bosch Institute of Clinical Pharmacology, Stuttgart, Germany

^5^ Departments of Clinical Pharmacology, Pharmacy and Biochemistry, University of Tübingen, Tübingen, Germany

*Share last authorship

Correspondence:

Professor Dr. Tobias Geisler,

Department of Cardiology,

University Hospital Tübingen,

Otfried‐Müller Str. 10,

72076 Tübingen,

Germany.

Email: tobias.geisler@med.uni-tuebingen.de

Submitted to BMC Cardiovascular Disorders

*Supplementary figure 2:* Comparison of individual miRNA expression levels stratified according to Δ LVEF%

*Supplementary figure 2a:* Volcano plot related to the analysis of individual miRNA expression levels stratified according to ∆ LVEF% <1 (n=7) or >1 (n=21). The x-axis presents fold change on a log_2_ scale (positive values represent a higher expression of miRNAs in ∆ LVEF% <1 compared to ∆ LVEF% >1, the y-axis displays -log_10_ p-values (blue dots above the green line (p=0.05) represent significantly differentially expressed miRNAs).

*Supplementary figure 2b:* Box plots comparing expression levels of miRNA 23b-3p and 29b-3p in patients with ∆ LVEF% <1, 1 and >1. p-values and corresponding fold changes (fc) are indicated on top of the boxes


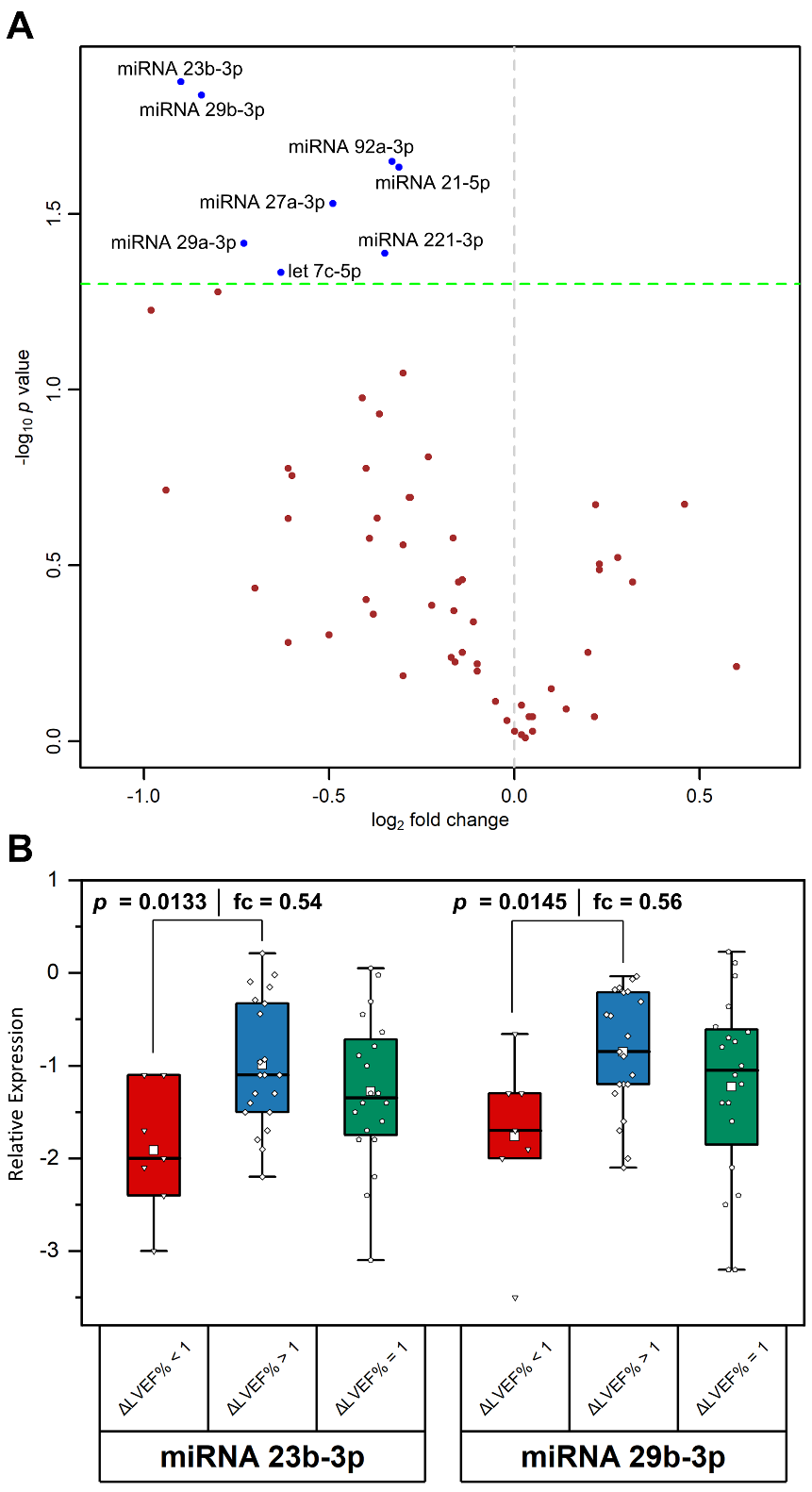

Supplement: Supplementary file 2 — Supplementary figure 2: Comparison of individual miRNA expression levels stratified according to Δ LVEF% [file 12872_2023_3362_MOESM2_ESM.docx]
