# Supplementary material for: Platelet miRNAs: differential expression in coronary artery disease and associations with course of left ventricular systolic function
Source: BMC Cardiovasc Disord. 2023 Jul 12;23:348. doi: 10.1186/s12872-023-03362-0 (PMC10339596; doi:10.1186/s12872-023-03362-0)

**Platelet miRNAs: Differential expression in coronary artery disease and associations with course of left ventricular systolic function**

Andreas Goldschmied^1^, Bernhard Drotleff^2^, Stefan Winter^3,4^, Elke Schaeffeler^3,4^, Matthias Schwab^4,5^, Meinrad Gawaz^1^, Tobias Geisler^1^* Dominik Rath^1*^

^1^ Department of Cardiology, University Hospital Tübingen, Tübingen, Germany

^2^ European Molecular Biology Laboratory, Heidelberg, Germany

^3^ University of Tübingen, Tübingen, Germany

^4^ Dr. Margarete‐Fischer‐Bosch Institute of Clinical Pharmacology, Stuttgart, Germany

^5^ Departments of Clinical Pharmacology, Pharmacy and Biochemistry, University of Tübingen, Tübingen, Germany

*Share last authorship

Correspondence:

Professor Dr. Tobias Geisler,

Department of Cardiology,

University Hospital Tübingen,

Otfried‐Müller Str. 10,

72076 Tübingen,

Germany.

Email: tobias.geisler@med.uni-tuebingen.de

Submitted to BMC Cardiovascular Disorders

*Supplementary figure 3*: Comparison of miRNA concentrations in platelets before and after activation with CRP. The y-axis represents relative miRNA expression, significant p-values and corresponding fold changes (fc) are indicated on top of the boxes.

*Supplementary figure 3a:* Box plots comparing expression levels of miRNAs 103a-3p, 30b-5p, 30c-5p, 185-5p, 140-3p and 221-3p in platelets before (non-act) and after (act) in vitro platelet activation in patients with MI (n=11).

*Supplementary figure 3b:* Box plots comparing expression levels of miRNAs 103a-3p, 30b-5p, 30c-5p, 185-5p, 140-3p and 221-3p in platelets before (non-act) and after (act) in vitro platelet activation in patients with CCS (n=10).

*Supplementary figure 3c:* Box plots comparing expression levels of miRNAs 103a-3p, 30b-5p, 30c-5p, 185-5p, 140-3p and 221-3p in platelets before (non-act) and after (act) in vitro platelet activation in controls (n=13).


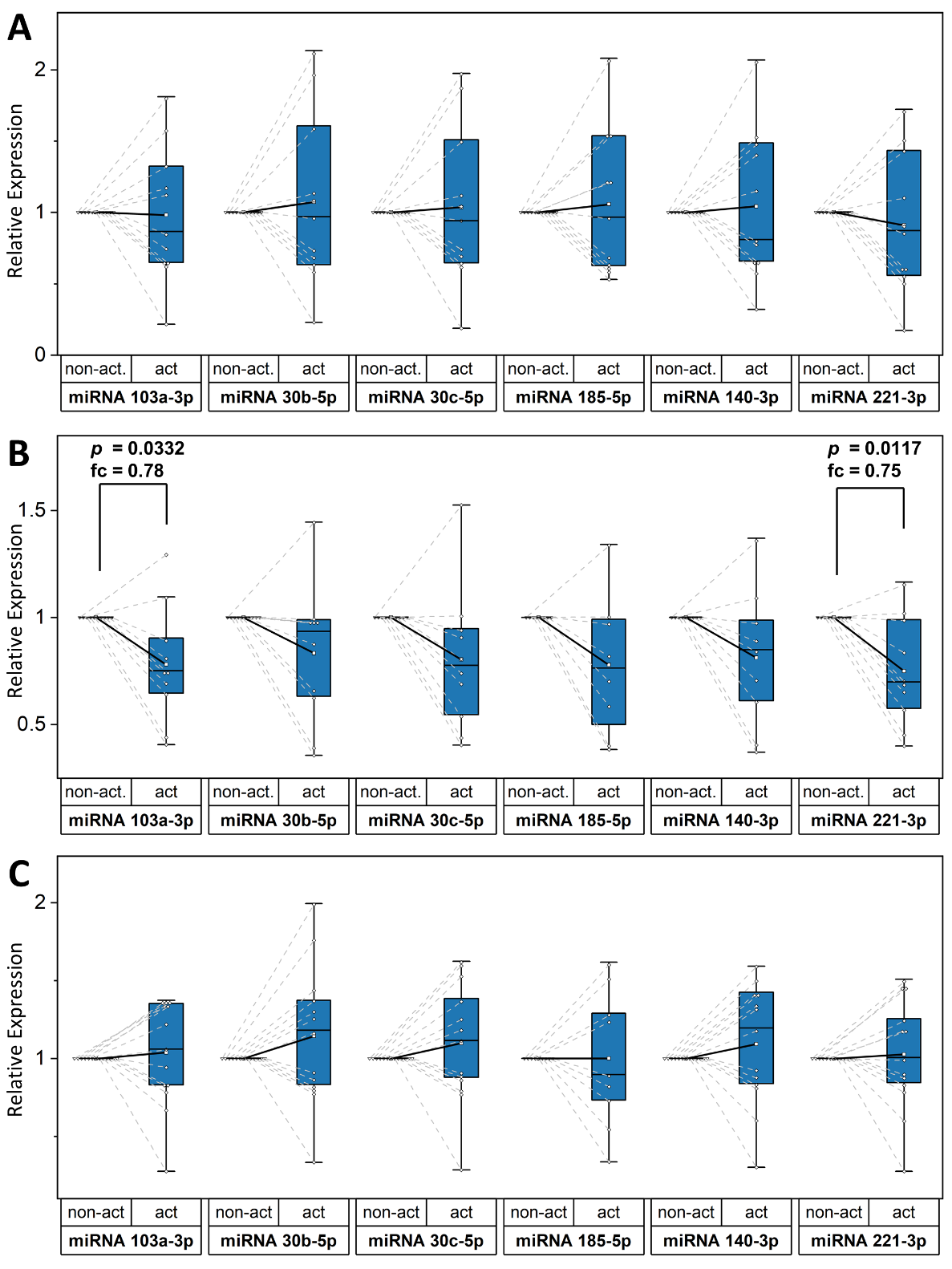

Supplement: Supplementary file 3 — Supplementary figure 3: Comparison of miRNA concentrations in platelets before and after activation with CRP. [file 12872_2023_3362_MOESM3_ESM.docx]
