# Supplementary material for: Platelet miRNAs: differential expression in coronary artery disease and associations with course of left ventricular systolic function
Source: BMC Cardiovasc Disord. 2023 Jul 12;23:348. doi: 10.1186/s12872-023-03362-0 (PMC10339596; doi:10.1186/s12872-023-03362-0)
Supplement: Supplementary file 4 — Supplementary table 1: 62 selected miRNAs [file 12872_2023_3362_MOESM4_ESM.docx]

**Platelet miRNAs: Differential expression in coronary artery disease and associations with course of left ventricular systolic function**

Andreas Goldschmied^1^, Bernhard Drotleff^2^, Stefan Winter^3,4^, Elke Schaeffeler^3,4^, Matthias Schwab^4,5^, Meinrad Gawaz^1^, Tobias Geisler^1^* Dominik Rath^1*^

^1^ Department of Cardiology, University Hospital Tübingen, Tübingen, Germany

^2^ European Molecular Biology Laboratory, Heidelberg, Germany

^3^ University of Tübingen, Tübingen, Germany

^4^ Dr. Margarete‐Fischer‐Bosch Institute of Clinical Pharmacology, Stuttgart, Germany

^5^ Departments of Clinical Pharmacology, Pharmacy and Biochemistry, University of Tübingen, Tübingen, Germany

*Share last authorship

Correspondence:

Professor Dr. Tobias Geisler,

Department of Cardiology,

University Hospital Tübingen,

Otfried‐Müller Str. 10,

72076 Tübingen,

Germany.

Email: tobias.geisler@med.uni-tuebingen.de

Submitted to BMC Cardiovascular Disorders

*Supplementary table 1:* 62 selected miRNAs

| **miRNA** | **Min** | **Max** | **Average** | **SD** |  | **miRNA** | **Min** | **Max** | **Average** | **SD** |
| --- | --- | --- | --- | --- | --- | --- | --- | --- | --- | --- |
| miRNA 1207-5p | -1.80 | 5.00 | 2.29 | ±1.79 |  | miRNA 17-5p | -1.20 | 0.90 | 0.00 | ±0.39 |
| miRNA 638 | -1.40 | 5.20 | 1.84 | ±1.87 |  | miRNA 30a-5p | -1.00 | 0.86 | -0.00 | ±0.44 |
| miRNA 206 | -1.10 | 4.30 | 1.31 | ±1.46 |  | miRNA 25-3p | -1.10 | 0.80 | -0.07 | ±0.36 |
| miRNA 208a-3p | -2.00 | 4.10 | 0.83 | ±1.33 |  | miRNA 92a-3p | -1.10 | 0.78 | -0.01 | ±0.38 |
| miRNA 539-5p | -2.20 | 3.30 | 0.65 | ±1.09 |  | miRNA 320c | -0.86 | 0.82 | -0.08 | ±0.38 |
| miRNA 20b-5p | -0.92 | 1.40 | 0.33 | ±0.46 |  | let7a5pp | -2.10 | 1.30 | -0.11 | ±0.80 |
| miRNA 93-5p | -1.00 | 1.20 | 0.32 | ±0.46 |  | miRNA 155-5p | -1.90 | 1.10 | -0.12 | ±0.60 |
| miRNA 146b-5p | -1.10 | 1.30 | 0.30 | ±0.47 |  | miRNA 223-3p | -1.60 | 0.80 | -0.13 | ±0.43 |
| let7c5p | -2.20 | 2.10 | 0.29 | ±0.82 |  | miRNA 21-5p | -1.10 | 1.10 | -0.17 | ±0.41 |
| miRNA 30c-5p | -0.87 | 1.20 | 0.27 | ±0.42 |  | miRNA 15a-5p | -3.20 | 1.50 | -0.18 | ±0.86 |
| miRNA 146b-3p | -1.60 | 1.30 | 0.25 | ±0.60 |  | miRNA 185-5p | -2.00 | 1.10 | -0.18 | ±0.49 |
| let7e5p | -1.60 | 1.60 | 0.23 | ±0.66 |  | miRNA 342-3p | -1.70 | 1.40 | -0.24 | ±0.72 |
| miRNA 133a-3p | -2.10 | 3.50 | 0.19 | ±1.05 |  | miRNA 125b-5p | -2.40 | 1.30 | -0.24 | ±0.73 |
| miRNA 103a-3p | -0.96 | 1.20 | 0.19 | ±0.40 |  | miRNA 320a | -1.30 | 0.86 | -0.27 | ±0.44 |
| miRNA 125a-5p | -2.00 | 1.80 | 0.16 | ±0.84 |  | miRNA 425-3p | -2.70 | 1.60 | -0.28 | ±0.71 |
| miRNA 24-3p | -1.10 | 1.30 | 0.16 | ±0.48 |  | miRNA 29c-3p | -2.00 | 0.99 | -0.30 | ±0.59 |
| miRNA 30b-5p | -1.00 | 0.93 | 0.16 | ±0.38 |  | miRNA 27a-3p | -1.50 | 0.53 | -0.31 | ±0.46 |
| miRNA 221-3p | -1.00 | 1.40 | 0.14 | ±0.43 |  | miRNA 26b-5p | -1.80 | 0.86 | -0.44 | ±0.56 |
| miRNA 22-5p | -1.50 | 1.40 | 0.14 | ±0.48 |  | miRNA 22-3p | -1.70 | 0.59 | -0.49 | ±0.49 |
| let7b5p | -2.10 | 2.40 | 0.12 | ±0.84 |  | miRNA 132-3p | -2.50 | 1.00 | -0.64 | ±0.82 |
| miRNA 16-5p | -1.30 | 1.40 | 0.12 | ±0.48 |  | miRNA 140-3p | -5.00 | 1.30 | -0.65 | ±1.04 |
| miRNA 451a | -2.00 | 2.40 | 0.11 | ±0.79 |  | miRNA 212-3p | -2.90 | 0.95 | -0.66 | ±0.75 |
| miRNA 452 | -3.50 | 3.50 | 0.09 | ±1.54 |  | miRNA 31-5p | -7.10 | 3.10 | -0.74 | ±1.79 |
| miRNA 126-3p | -1.90 | 1.20 | 0.09 | ±0.50 |  | miRNA 195-5p | -3.30 | 2.40 | -0.78 | ±1.02 |
| miRNA 378a-3p | -1.80 | 1.40 | 0.07 | ±0.59 |  | miRNA 29b-3p | -3.50 | 0.91 | -1.00 | ±0.92 |
| miRNA 191-5p | -1.30 | 1.10 | 0.06 | ±0.46 |  | miRNA 23a-3p | -3.70 | 0.87 | -1.06 | ±0.86 |
| miRNA 15b-5p | -1.10 | 1.10 | 0.06 | ±0.39 |  | miRNA 199a-5p | -4.30 | 0.80 | -1.08 | ±0.91 |
| miRNA 20a | -2.20 | 2.40 | 0.06 | ±0.98 |  | miRNA 23b-3p | -3.10 | 0.42 | -1.24 | ±0.82 |
| miRNA 1-3p | -5.20 | 4.10 | 0.04 | ±1.83 |  | miRNA 133b-3p | -9.70 | 4.60 | -1.29 | ±3.23 |
| miRNA 423-5p | -1.10 | 1.00 | 0.02 | ±0.34 |  | miRNA 886-3p | -5.30 | 3.40 | -2.13 | ±1.88 |
| miRNA 29a-3p | -2.50 | 2.30 | 0.02 | ±0.90 |  | miRNA 34a | -4.00 | 3.80 | -3.79 | ±3.41 |
