# Supplementary material for: Platelet miRNAs: differential expression in coronary artery disease and associations with course of left ventricular systolic function
Source: BMC Cardiovasc Disord. 2023 Jul 12;23:348. doi: 10.1186/s12872-023-03362-0 (PMC10339596; doi:10.1186/s12872-023-03362-0)
Supplement: Supplementary file 5 — Supplementary table 2: Significantly altered miRNAs and suggested cardiovascular effects [file 12872_2023_3362_MOESM5_ESM.docx]

**Platelet miRNAs: Differential expression in coronary artery disease and associations with course of left ventricular systolic function**

Andreas Goldschmied^1^, Bernhard Drotleff^2^, Stefan Winter^3,4^, Elke Schaeffeler^3,4^, Matthias Schwab^4,5^, Meinrad Gawaz^1^, Tobias Geisler^1^* Dominik Rath^1*^

^1^ Department of Cardiology, University Hospital Tübingen, Tübingen, Germany

^2^ European Molecular Biology Laboratory, Heidelberg, Germany

^3^ University of Tübingen, Tübingen, Germany

^4^ Dr. Margarete‐Fischer‐Bosch Institute of Clinical Pharmacology, Stuttgart, Germany

^5^ Departments of Clinical Pharmacology, Pharmacy and Biochemistry, University of Tübingen, Tübingen, Germany

*Share last authorship

Correspondence:

Professor Dr. Tobias Geisler,

Department of Cardiology,

University Hospital Tübingen,

Otfried‐Müller Str. 10,

72076 Tübingen,

Germany.

Email: tobias.geisler@med.uni-tuebingen.de

Submitted to BMC Cardiovascular Disorders

*Supplementary table 2:* Significantly altered miRNAs and suggested cardiovascular effects

| miRNA | Cardiovascular effects | Authors |
| --- | --- | --- |
| miRNA 103 | Promotes cardiomyocyte necrosis through FADD activation | Jian-Xun Wang et al. *Circ Res*. 2015 |
|  | Endothelial Cell MiR-103 induces endothelial inflammation and atherosclerosis by suppression of KLF4 | Hartmann et al. Nature Communications. 2015 |
| miRNA 155 | Associated with Inhibition of murine megakaryopoiesis | Georgantas et al. Proc Natl Acad Sci U S A. 2007 |
|  | Promotes atherosclerosis via endothelial cell activation | Virtue et al. Journal of Biological Chemistry. 2017 |
|  | Downregulation in plasma of CAD patients compared to healthy controls | Fichtlscherer et al. Circ Res. 2010 |
|  | Downregulation in PBMCs of ACS patients compared to stable CAD | Yao et al. Cell Mol Immunol. 2011 |
|  | Upregulation in plasma associated with coronary slow flow | Su et al. Dis Markers. 2018 |
