# Supplementary material for: Platelet miRNAs: differential expression in coronary artery disease and associations with course of left ventricular systolic function
Source: BMC Cardiovasc Disord. 2023 Jul 12;23:348. doi: 10.1186/s12872-023-03362-0 (PMC10339596; doi:10.1186/s12872-023-03362-0)
Supplement: Supplementary file 6 — Supplementary: Platelet isolation Protocol [file 12872_2023_3362_MOESM6_ESM.docx]

**Platelet miRNAs: Differential expression in coronary artery disease and associations with course of left ventricular systolic function**

Andreas Goldschmied^1^, Bernhard Drotleff^2^, Stefan Winter^3,4^, Elke Schaeffeler^3,4^, Matthias Schwab^4,5^, Meinrad Gawaz^1^, Tobias Geisler^1^* Dominik Rath^1*^

^1^ Department of Cardiology, University Hospital Tübingen, Tübingen, Germany

^2^ European Molecular Biology Laboratory, Heidelberg, Germany

^3^ University of Tübingen, Tübingen, Germany

^4^ Dr. Margarete‐Fischer‐Bosch Institute of Clinical Pharmacology, Stuttgart, Germany

^5^ Departments of Clinical Pharmacology, Pharmacy and Biochemistry, University of Tübingen, Tübingen, Germany

*Share last authorship

Correspondence:

Professor Dr. Tobias Geisler,

Department of Cardiology,

University Hospital Tübingen,

Otfried‐Müller Str. 10,

72076 Tübingen,

Germany.

Email: tobias.geisler@med.uni-tuebingen.de

Submitted to BMC Cardiovascular Disorders

*Supplementary: Platelet isolation Protocol*

**Platelet isolation Protocol**

60ml of arterial whole blood was taken in three 20ml syringes during coronary angiography and immediately transferred to the in-house scientific laboratory. There, the blood was pooled in a ratio of 1:4 with [Acid-Citrate-Dextrose (ACD)](https://de.wikipedia.org/wiki/Acid-Citrate-Dextrose) anticoagulant buffer, distributed into 15ml falcons and centrifuged at 330xg for 10 minutes at room temperature without breaks. Using a Pasteur pipette, the platelet rich plasma was pooled at a ratio 1:2 with Tyrodes buffer pH 6.5 in a 50ml falcon and redistributed to 15ml falcons. Next, the solution was centrifuged at room temperature without breaks at 240xg for 10 minutes. The supernatant was transferred to new 15ml falcons and a 50µl aliquot was tested for potential leukocyte contamination using a clinical hematology analyzer (Sysmex). If leukocyte contamination could be excluded, the samples were centrifuged at room temperature without breaks at 430xg for 10 minutes. The supernatant was discarded and the platelet pellet re-suspended in Tyrodes pH 6.5 buffer. A 50µl aliquot was used to estimate platelet count using the clinical hematology analyzer. Finally, the sample was split into two aliquots and transferred to 1.5ml microcentrifuge tubes and centrifuged at 420xg for 5 minutes at 4° Celsius. Afterwards, the samples were labeled and stored at -80° Celsius.
